# Survival Modelling Using Machine Learning and Immune–Nutritional Profiles in Advanced Gastric Cancer on Home Parenteral Nutrition

**DOI:** 10.3390/nu17152414

**Published:** 2025-07-24

**Authors:** Konrad Matysiak, Aleksandra Hojdis, Magdalena Szewczuk

**Affiliations:** 1 Centre for Intestinal Failure, Poznan University of Medical Sciences, 60-355 Poznań, Poland; 2 Department of Gastroenterology, Poznan University Hospital, 60-355 Poznań, Poland; aleksandra.hojdis@gmail.com (A.H.); magdaszewczuk@wp.pl (M.S.); 3 Department of Internal Medicine, Poznan University of Medical Sciences, 60-355 Poznań, Poland

**Keywords:** machine learning in nutritional science, personalised nutrition, predictive modelling in nutrition, nutritional biomarkers, immune–inflammatory biomarkers, random survival forest

## Abstract

**Background/Objectives:** Patients with stage IV gastric cancer who develop chronic intestinal failure require home parenteral nutrition (HPN). This study aimed to evaluate the prognostic relevance of nutritional and immune–inflammatory biomarkers and to construct an individualised survival prediction model using machine learning techniques. **Methods:** A secondary analysis was performed on a cohort of 410 patients with TNM stage IV gastric adenocarcinoma who initiated HPN between 2015 and 2023. Nutritional and inflammatory indices, including the Controlling Nutritional Status (CONUT) score and lymphocyte-to-monocyte ratio (LMR), were assessed. Independent prognostic factors were identified using Cox proportional hazards models. A Random Survival Forest (RSF) model was constructed to estimate survival probabilities and quantify variable importance. **Results:** Both the CONUT score and LMR were independently associated with overall survival. In multivariate analysis, higher CONUT scores were linked to increased mortality risk (HR = 1.656, 95% CI: 1.306–2.101, *p* < 0.001), whereas higher LMR values were protective (HR = 0.632, 95% CI: 0.514–0.777, *p* < 0.001). The RSF model demonstrated strong predictive accuracy (C-index: 0.985–0.986) and effectively stratified patients by survival risk. The CONUT score exerted the greatest prognostic influence, with the LMR providing additional discriminatory value. A gradual decline in survival probability was observed with an increasing CONUT score and a decreasing LMR. **Conclusions:** The application of machine learning to immune–nutritional data offers a robust tool for predicting survival in patients with advanced gastric cancer requiring HPN. This approach may enhance risk stratification, support individualised clinical decision-making regarding nutritional interventions, and inform treatment intensity adjustment.

## 1. Introduction

Gastric cancer remains one of the most commonly diagnosed malignancies worldwide and represents the third leading cause of cancer-related mortality [1]. Patients with stage IV disease, as classified by the TNM system, exhibit particularly poor prognoses. In this group, tumour progression frequently leads to metabolic and nutritional complications. In many cases, parenteral nutritional therapy constitutes the only feasible form of medical management.

Progressive malnutrition is a major determinant of survival in advanced gastric cancer and has been consistently associated with reduced overall survival [2]. Metabolic disturbances contribute significantly to the development of malnutrition and cachexia, reflecting the role of metabolic dysregulation in disease progression [3]. Systemic inflammation resulting from tumour–host interactions is characterised by elevated levels of pro-inflammatory cytokines, including tumour necrosis factor α, interleukin-1, and interleukin-6. These changes are associated with anorexia, skeletal muscle breakdown, and enhanced lipolysis, leading to catabolism and reduced treatment efficacy [4]. Chronic intestinal failure further impairs metabolic status. Disrupted intestinal transit and diminished absorption of macronutrients, fluids, and electrolytes exacerbate nutritional deficiencies. In patients with chronic intestinal failure, parenteral nutrition constitutes the only effective and clinically justified form of nutritional support [5].

Emerging evidence indicates a strong association between tumour biology, the surrounding microenvironment, and systemic inflammatory responses [6]. Peripheral blood biomarkers reflecting nutritional status [7] and immune activity [8] may offer valuable prognostic insights. Their application facilitates risk stratification and supports the individualisation of treatment approaches. Nutritional parameters, such as total lymphocyte count (TLC), have been identified as independent prognostic factors for overall survival in patients with gastric cancer [9]. The preoperative Controlling Nutritional Status (CONUT) score has been demonstrated to be a reliable prognostic indicator in patients undergoing gastrectomy [10] and those receiving adjuvant therapy [11]. Malnutrition, evaluated based on reduced skeletal muscle mass and a low prognostic nutritional index (PNI), has been associated with poorer postoperative overall survival outcomes [12]. The Naples Prognostic Score (NPS) has been recognised as a valid tool for assessing survival in patients with recurrent gastric cancer occurring five or more years after gastrectomy [7]. Inflammatory markers, including the neutrophil-to-lymphocyte ratio (NLR), have been linked to unfavourable prognosis in patients with unresectable or recurrent gastric cancer [13]. Wu et al. reported that elevated systemic immune–inflammation index (SII) levels were associated with reduced overall survival and lower five-year survival rates [14]. Similarly, a low lymphocyte-to-monocyte ratio (LMR) in post-gastrectomy patients has been identified as an independent predictor of decreased overall and recurrence-free survival [15]. Previous analyses have confirmed the prognostic value of nutritional biomarkers, particularly in patients with advanced gastric cancer receiving home parenteral nutrition due to tumour-related intestinal failure [16]. However, despite their validated prognostic value, these analyses do not enable precise individualisation of survival prediction. The integration of machine learning methods into survival analysis has facilitated more accurate risk stratification and improved individualised prognostication [17,18].

Random Survival Forest (RSF), a machine learning technique, enables the identification of complex interactions among prognostic variables and their relationship with patient survival time. RSF estimates individual survival functions by aggregating outputs from multiple decision trees, providing more accurate survival predictions [19]. RSF-based models have demonstrated utility in predicting treatment outcomes and survival in patients with gastric cancer [20,21]. In contrast to parametric methods, which require specific assumptions regarding the hazard function, RSF operates without such constraints, making it a flexible tool for modelling complex prognostic relationships [19]. It facilitates the analysis of moderately sized biomedical datasets with a focus on survival outcomes and performs well even in the presence of correlated prognostic variables and partially incomplete data. The algorithm’s random selection of subsets of features and observations reduces tree correlation, enhancing model robustness and limiting the risk of overfitting [21].

In this context, the present study aimed to evaluate the relationship between nutritional and immuno-inflammatory biomarkers and the estimation of individual survival functions in patients with stage IV gastric cancer receiving home parenteral nutrition secondary to chronic intestinal failure.

## 2. Materials and Methods

### 2.1. Study Population

This secondary analysis was conducted using a cohort of patients who commenced home parenteral nutrition between 1 January 2015 and 31 December 2023. The observation period concluded on 31 July 2024. Only patients with confirmed stage IV gastric cancer according to the TNM classification were eligible for inclusion. Full details of the study population have been reported previously [16]; key eligibility criteria are briefly summarised below. Inclusion criteria comprised TNM stage IV gastric cancer, chronic intestinal failure precluding oral intake, and initiation of HPN. Exclusion criteria were the presence of hepatic metastases, obstructive jaundice, refractory malignant ascites, catheter-related bloodstream infection during HPN, malignant gastric outlet obstruction, and lack of informed consent. All clinical and laboratory data were complete across the analysed cohort. No imputation methods were required.

The primary objective of this analysis was to identify clinical and demographic factors, including selected biomarkers of nutritional status and immune response, that are significantly associated with overall survival in patients with TNM stage IV gastric cancer initiating HPN due to chronic intestinal failure. The secondary objective was to develop a survival prediction model using machine learning techniques. To this end, the Random Survival Forest model was applied to estimate individual survival functions, determine variable importance, and support patient risk stratification.

### 2.2. Data Collection

Nutritional and immuno-inflammatory status was assessed using a panel of validated indices and laboratory parameters obtained on the day of hospital admission for home parenteral nutrition qualification.

- The Controlling Nutritional Status score [22] was calculated based on the following:
  - Serum albumin (g/L);
  - Total cholesterol (mg/dL);
  - Total lymphocyte count.

According to the CONUT index, patients were classified as follows:

- 0–1 points: well-nourished;
- 2–4 points: mildly malnourished;
- 5–8 points: moderately malnourished;
- 9–12 points: severely malnourished.

- The prognostic nutritional index [23] considered the following:
  - Serum albumin (g/dL);
  - Total lymphocyte count.

The PNI was calculated using the following formula: (10 × albumin [g/dL]) + (0.005 × lymphocyte count [/mm^3^]).

- The Naples Prognostic Score [24] combined the following:
  - Serum albumin (g/L);
  - Total cholesterol (mg/dL);
  - Neutrophil-to-lymphocyte ratio;
  - Lymphocyte-to-monocyte ratio.

Each parameter was scored 0 or 1. The final NPS was calculated as the sum of these individual scores.

The NPS classification was as follows:

- Score 0: low risk;
- Score 1–2: intermediate risk;
- Score 3–4: high risk.

- The Lymphocyte-to-monocyte ratio [25] was calculated.

The LMR was calculated as the ratio of the absolute lymphocyte count to the absolute monocyte count (×10^9^/L).

- Systemic Immune–inflammation Index [26]. The SII was calculated using the following formula: (platelet count × neutrophil count)/lymphocyte count. All counts were expressed in ×10^9^/L.
- The neutrophil-to-lymphocyte ratio was calculated [27].

The NLR was calculated as the ratio of the absolute neutrophil count to the absolute lymphocyte count, both expressed in ×10^9^/L.

- The total lymphocyte count was calculated [28].

The TLC was calculated by multiplying the total white blood cell count by the lymphocyte percentage and dividing by 100 (expressed as ×10^9^/L). It was used as a component of the CONUT and PNI indices and as an individual immune–nutritional marker.

- Body mass index was defined as weight in kilograms divided by the square of height in metres (kg/m^2^).

Biochemical assessments were conducted in a certified university hospital laboratory, following standardised protocols and good laboratory practice. The evaluation included the following parameters:

- Complete blood count;
- White blood cell count (×10^9^/L);
- Lymphocyte count (×10^9^/L);
- Lymphocyte percentage (%);
- Monocyte (×10^9^/L), absolute;
- Neutrophil count (×10^9^/L);
- Platelet count (×10^9^/L);
- Total protein (g/dL);
- Serum albumin (g/L);
- Total cholesterol (mg/dL).

### 2.3. Statistical Analyses

Variables exhibiting significant skewness were transformed using cut-off values derived from receiver operating characteristic (ROC) curves and the Youden index. Binary and categorical variables were analysed without transformation. Survival analysis was performed using Cox proportional hazards models, beginning with univariate analysis to assess the association between individual variables and overall survival. Variables found to be statistically significant in the univariate analysis were subsequently entered into the multivariate Cox model to evaluate their independent prognostic value. Model performance was evaluated using regression coefficients, hazard ratios (HR = exp(coef)), and 95% confidence intervals. Collinearity was assessed using Pearson correlation coefficients, with a threshold of |r| > 0.6 indicating significant correlation. Additionally, the Variance Inflation Factor (VIF) was calculated, with VIF > 5 considered indicative of multicollinearity. Kaplan–Meier curves were used to visualise survival across CONUT and LMR groups, with comparisons performed using the log-rank test. To control for multiple comparisons and minimise the risk of false positive findings, a significance threshold of *p* < 0.001 was applied throughout the analysis.

### 2.4. Random Survival Forest—Methodology and Implementation

A Random Survival Forest model was implemented to predict overall survival while handling right-censored data. The dataset was split into a training set (80%) and a test set (20%), ensuring balanced representation of prognostic variables. Each tree in the forest was constructed using a random subset of the data and a randomly selected subset of features (maximum number of features = 50%) to determine optimal splits. Splitting was performed using the log-rank statistic to ensure survival-based node division. Hyperparameter tuning was conducted during model development, with final values set to number of estimators = 400, minimum number of samples required to split an internal node = 10, and minimum number of samples required to be at a leaf node = 5. To evaluate predictive performance, 5-fold cross-validation and 1000 bootstrap iterations were employed. Harrell’s Concordance Index (C-index) was computed across validation folds to assess the model’s ability to correctly rank survival times. Additionally, the Brier Score was used to evaluate the calibration of predicted survival probabilities. All analyses were conducted using Python version 3.12.0, utilising the Lifelines (v0.30.0), Statsmodels (v0.14.4), Scikit-survival (v0.23.1), and Scikit-learn (1.6.1) libraries. Survival curves were generated and visualised using Matplotlib (v3.10.3).

## 3. Results

### 3.1. Patient Characteristics

The analysed cohort comprised 410 patients (153 females and 257 males) aged 28 to 93 years, all with histologically confirmed gastric adenocarcinoma stage IV according to the TNM classification. The underlying cause of chronic intestinal failure was malignant mechanical obstruction in all cases. The median overall survival was 195 days (interquartile range: 285), ranging from 14 to 1262 days. During the study period, the mean daily volume of administered parenteral nutrition was 1721 ± 532 mL, providing an average of 28.6 ± 6.5 kcal/kg.

Prior to the initiation of parenteral nutrition, 74 patients (18%) underwent palliative gastrectomy. A total of 170 patients (40%) received concurrent 5-fluorouracil-based palliative chemotherapy during home parenteral nutrition. Stratified nutritional and inflammatory indices used for prognostic modelling are presented in Table 1.

### 3.2. ROC Analysis and Cut-Off Values

Receiver operating characteristic (ROC) curve analysis was performed for four prognostic biomarkers (PNI, LMR, NLR, SII) to determine optimal cut-off values using Youden’s test, which maximises the difference between sensitivity and specificity. Based on these thresholds, patients were stratified into high- and low-risk groups, and the biomarkers were transformed into categorical variables for subsequent Cox regression survival analysis.

Among the biomarkers, the PNI demonstrated the highest AUC (0.744), indicating strong predictive ability. Its cut-off value provided high sensitivity (81.8%) and moderate specificity (58.5%), making it a key tool for identifying patients with a worse prognosis. The LMR showed balanced sensitivity (68.0%) and specificity (64.3%), while the NLR exhibited lower sensitivity (51.7%) but higher specificity (78.0%). The findings of the ROC curve analysis are summarised in Table 2.

**Figure 1:**
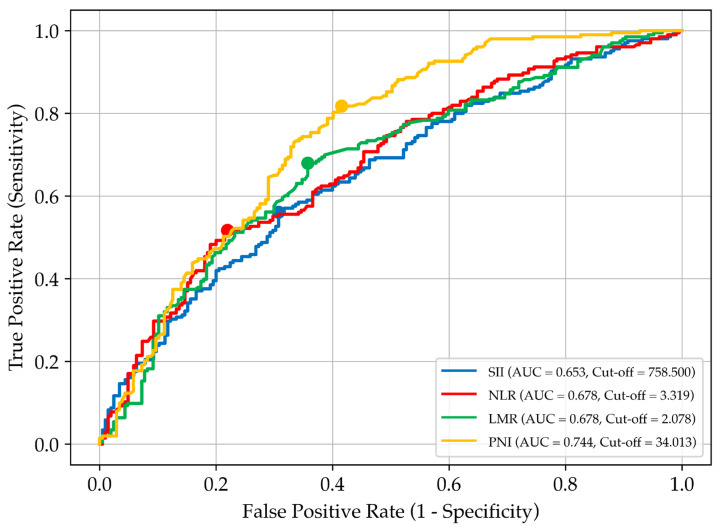
Receiver operating characteristic (ROC) curves for selected prognostic biomarkers used to assess overall survival in patients with stage IV gastric cancer receiving home parenteral nutrition. Each curve represents a different biomarker: SII, systemic immune–inflammation index; NLR, neutrophil-to-lymphocyte ratio; LMR, lymphocyte-to-monocyte ratio; and PNI, prognostic nutritional index. The area under the curve (AUC) reflects each biomarker’s discriminatory ability. Cut-off values were determined using Youden’s index and are marked by coloured dots on the corresponding curves.

Figure 1 illustrates the determination of optimal biomarker thresholds based on the ROC analysis and Youden’s test. 

### 3.3. Cox Regression: Univariate and Multivariate Analysis Results

Univariate Cox regression analysis was used to assess the impact of each variable on patient survival. The results showed that all biological markers were significantly associated with mortality risk, while sex, age, and BMI were not statistically significant and were excluded from further analysis.

Among the biomarkers, CONUT and LMR exhibited the strongest effects, with hazard ratios (HRs) of 2.110 and 0.487, respectively (*p* < 0.001). A higher CONUT score was associated with shorter survival, while higher LMR levels correlated with prolonged survival.

In the multivariate analysis, CONUT remained a strong predictor of poorer prognosis, although its effect was slightly reduced after adjustment (HR = 1.656, 95% CI: 1.306–2.101, *p* < 0.001). The persistent statistical significance (*p* < 0.001) confirms that its association with survival is robust. Similarly, the LMR remained an independent protective factor, with an adjusted HR of 0.632 (95% CI: 0.514–0.777, *p* < 0.001). An HR below 1 indicates a protective effect, with higher LMR levels associated with longer survival. The assessment of the model’s coefficient stability and its biological relevance is summarised in Table 3.

### 3.4. Collinearity Assessment

Potential multicollinearity was evaluated using the correlation matrix and VIF values to ensure model accuracy and reliability. The results showed that VIF values for all variables were below 5, indicating low multicollinearity. The correlation matrix confirmed the absence of strong relationships between variables, supporting the model’s stability and robustness. Assessment of multicollinearity using Pearson’s correlation and VIF is presented in Table 4.

### 3.5. Survival Analysis with Random Survival Forest

**Figure 2:**
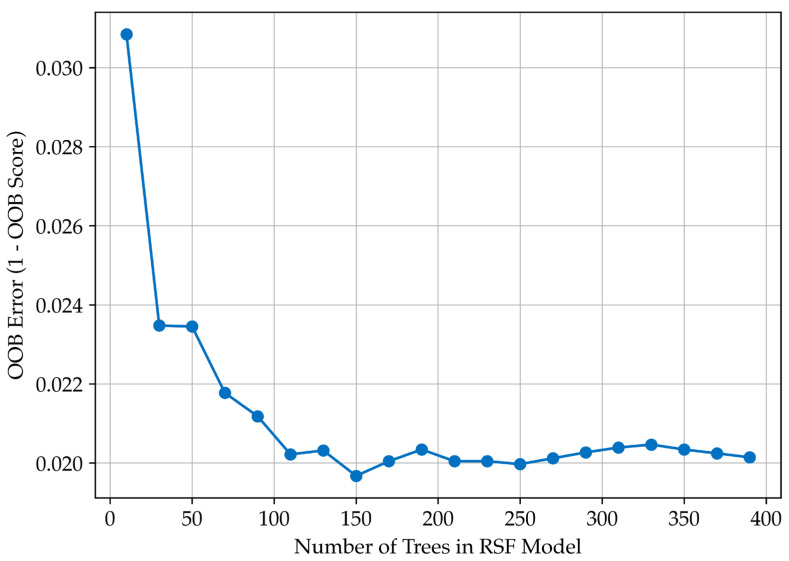
Random Survival Forest model performance. Relationship between the number of trees and out-of-bag (OOB) error, demonstrating model stabilisation at approximately 150–200 trees.

Cox regression identified CONUT and LMR as key prognostic biomarkers. These variables were used as inputs for a Random Survival Forest (RSF) model to estimate patient survival while accounting for right-censored data. The RSF model achieved high predictive performance, with the OOB error decreasing from 0.0308 (10 trees) to ~0.0200 at 150–200 trees, beyond which further improvement was negligible. The C-index remained consistently high (0.985–0.986), confirming the model’s strong ability to distinguish survival outcomes. These results indicate that the RSF model generalises well to unseen data and is not prone to overfitting (Figure 2).

The RSF model effectively stratifies patients by survival risk. Feature importance analysis showed that CONUT (0.226) had a much stronger impact on survival prediction than LMR (0.022), highlighting the prognostic value of nutritional status over the lymphocyte-to-monocyte ratio.

To assess the model’s calibration, the Brier Score was calculated, which measures how closely predicted survival probabilities match actual outcomes. The mean Brier Score from 5-fold cross-validation was 0.152 ± 0.011 (95% CI: 0.130–0.162), suggesting well-calibrated predictions with strong generalisability. Survival probabilities estimated using the Random Survival Forest model at different time points based on CONUT and LMR categories are presented in Table 5. This table summarises the predictive outputs generated by the model, illustrating stratified survival estimates across nutritional and immune–inflammatory groups.

### 3.6. Kaplan–Meier Survival Curves and Log-Rank Test for Survival Differences

**Figure 3:**
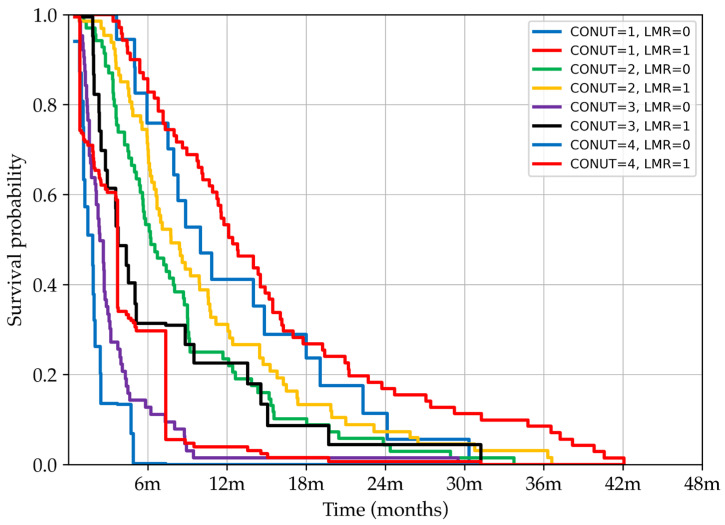
Kaplan–Meier survival curves showing the probability of survival over time stratified by combined CONUT, Controlling Nutritional Status, and LMR, lymphocyte-to-monocyte ratio, categories. Patients were grouped based on increasing CONUT scores (from 1 to 4) and dichotomised LMR values (high = 1, low = 0). The curves illustrate how the combination of nutritional status and immune–inflammatory response influences survival outcomes, with distinct prognostic separation observed between subgroups.

Kaplan–Meier survival curves stratified according to CONUT and LMR categories are displayed in Figure 3. The most significant survival differences occur within the first 12–24 months, confirming that the RSF model is most effective during this period.

Survival comparisons across patient groups stratified by CONUT and LMR were conducted using the log-rank test. Comparisons within the same nutritional category (e.g., CONUT = 1, LMR = 0 vs. CONUT = 1, LMR = 1; CONUT = 2, LMR = 0 vs. CONUT = 2, LMR = 1; CONUT = 4, LMR = 0 vs. CONUT = 4, LMR = 1) did not reach statistical significance, suggesting that isolated changes in LMR are not a primary determinant of survival when nutritional status is stable. In contrast, comparisons between adjacent CONUT categories (e.g., CONUT = 1, LMR = 1 vs. CONUT = 2, LMR = 0; CONUT = 2, LMR = 1 vs. CONUT = 3, LMR = 0; CONUT = 3, LMR = 1 vs. CONUT = 4, LMR = 0) yielded highly significant differences (*p* < 0.001), underscoring the dominant prognostic effect of worsening nutritional status. Importantly, within the CONUT = 3 subgroup, the survival difference between LMR categories (0 vs. 1) reached statistical significance (*p* < 0.001), indicating that under conditions of advanced malnutrition, the immunological factor represented by the LMR adds meaningful prognostic value. Collectively, these results highlight the hierarchical prognostic contribution of the studied biomarkers, with CONUT emerging as the primary predictor and LMR acting as a complementary factor, particularly in severely malnourished patients.

## 4. Discussion

This study aimed to assess the relationship between nutritional and immune-related biomarkers and individual survival estimation in patients with stage IV gastric cancer, classified according to the TNM system, who required home parenteral nutrition due to chronic intestinal failure. Based on the conducted analysis, the prognostic significance and potential clinical utility of selected indicators were discussed.

The analysis was conducted in two stages. Initially, Cox regression identified the CONUT and LMR indices as independent prognostic factors for overall survival. Subsequently, the RSF algorithm was applied to model nonlinear relationships between biomarker values and survival time. An RSF, as an extension of traditional survival analysis methods, enables prediction based on an ensemble of decision trees [19]. The model demonstrated high predictive accuracy, with CONUT receiving a higher prognostic weight than LMR, emphasising its relevance in survival analysis among patients receiving HPN. 

The CONUT score, based on three routinely measured biochemical parameters—total cholesterol, serum albumin concentration, and total lymphocyte count—provides an objective assessment of both nutritional status and immune competence. Cholesterol was initially incorporated into the CONUT score as a marker of caloric depletion [22]. Subsequent studies demonstrated that abnormalities in cholesterol metabolism, including excessive biosynthesis and impaired transport, are significantly associated with cancer progression and poor prognosis [29]. The relationship between peripheral blood cholesterol levels and survival outcomes is also supported by data from a large cohort study, where higher total cholesterol levels correlated with a reduced risk of gastric cancer, particularly among men, with a similar trend observed in women [30]. In the CONUT score, albumin serves as an indicator of protein reserves [22]. Albumin contributes to modulation of the inflammatory response through its antioxidant properties and scavenging functions, potentially reducing the release of pro-inflammatory cytokines [31]. Preoperative hypoalbuminaemia has been linked to poor survival outcomes in patients with gastric cancer [32] and significantly worsens prognosis following radical gastrectomy [33]. Pre-treatment total lymphocyte count reflects antitumour immunity, with lower counts associated with poorer survival and a reduced capacity to inhibit tumour progression [34]. The prognostic utility of the CONUT score has been confirmed in retrospective studies involving patients with gastric cancer [35], including its relevance in evaluating postoperative complications [36], shorter survival after surgery [10], and response to adjuvant therapy [37].

The observed survival differences across CONUT categories, identified using the log-rank test, reinforce the central prognostic role of nutritional status in patients with advanced gastric cancer receiving home parenteral nutrition. While isolated variations in the LMR were generally non-significant, the LMR provided meaningful prognostic value specifically within the subgroup of patients with more advanced malnutrition (CONUT = 3). This suggests a hierarchical interplay, in which malnutrition exerts a dominant influence on survival, while immuno-inflammatory factors become relevant modulators in settings of severe nutritional compromise. These findings highlight the importance of combining nutritional and inflammatory markers for refined risk stratification and suggest that future therapeutic strategies should prioritise nutritional optimisation alongside inflammation control to improve patient outcomes.

In this cohort, the LMR demonstrated a weaker association with survival than CONUT but served as a significant complementary marker. Incorporating the LMR into the model improved the precision of risk stratification and allowed for a more accurate prognosis assessment, particularly in patients with mild or moderate malnutrition as classified by CONUT. With its balanced sensitivity and specificity, the LMR aids in identifying both high- and low-risk patients and reduces the likelihood of misclassification by minimising false positives and negatives. The prognostic mechanism underlying the LMR may be linked to the role of lymphocytes in immune surveillance, including their capacity to recognise malignant cells and induce apoptosis [38], and the protumour functions of monocytes, which contribute to tumour development and angiogenesis [39]. A study by Feng et al. demonstrated that a high monocyte count and low lymphocyte count were independently associated with poor prognosis in gastric cancer [40]. The independent prognostic value of the pre-treatment LMR has been confirmed in patients undergoing gastrectomy [41,42] and is associated with increased mortality risk and shorter overall survival [43]. The ability of the LMR to discriminate between prognostic subgroups supports more precise individualisation of therapeutic strategies and more effective monitoring of outcomes [44]. Although the LMR was dichotomised in the present analysis to support clinical applicability, we acknowledge that such an approach may overlook subtle gradations in prognostic risk. Future studies may benefit from modelling the LMR and other biomarkers as continuous variables within a multidimensional input space, thereby enabling more nuanced and probabilistic risk estimation.

The application of machine learning approaches, including RSF, allows for more comprehensive risk stratification [17], improved prediction of individual survival times, and integration of diverse biological parameters into prognostic models [45]. This methodology has previously been applied to estimate the risk of gastric cancer progression [46], assess treatment efficacy [18], optimise therapeutic strategies, and monitor patients at high risk of poor outcomes [47]. The RSF model presented in this study estimated survival probability across time intervals, accounting for interactions between CONUT and LMR. It facilitated risk stratification and identification of subgroups in which the combination of CONUT and LMR exerted the greatest prognostic impact, demonstrating a systematic decline in survival probability with increasing CONUT and decreasing LMR values.

The strength of the present study lies in the application of machine learning methods, enabling the integration of complex data, risk stratification, and survival prediction. The analysis was based on objective biochemical markers, which helped mitigate potential bias associated with the retrospective design. The developed survival prediction model has important clinical relevance for identifying patients eligible for home parenteral nutrition and for recognising groups with an increased risk of malnutrition and poor survival outcomes.

One of the main limitations of this study is the low representation of severely malnourished patients, which may affect the model’s stability and precision. The small sample size in this subgroup increases variance and limits the statistical power to detect significant associations. In the RSF model, this may result in less stable survival function estimates and reduced accuracy in assessing variable importance. Consequently, findings related to patients with the highest CONUT scores should be interpreted cautiously and validated in larger cohorts. Due to the relatively small number of individuals within this high-risk subgroup, the model’s predictive reliability for this category may be limited, and future studies involving more severely malnourished patients are needed to refine and confirm these findings. While the sample size of 410 patients allowed for meaningful survival modelling, the application of machine learning models in moderately sized clinical datasets remains inherently challenging. To mitigate the risk of overfitting, the model was internally validated using repeated five-fold cross-validation and bootstrapping. Nonetheless, future studies with larger and independent cohorts are warranted to confirm these findings and further validate the model’s stability. The absence of external validation represents a significant limitation, as both the training and testing datasets were derived from the same population, which may limit the generalisability of the results to broader patient populations. Although five-fold cross-validation and bootstrapping were performed to mitigate overfitting and evaluate the internal robustness of the model, validation using an independent external cohort would provide a more definitive assessment of model generalisability and ensure its applicability across broader clinical settings.

The predictive outputs generated by this analysis underscore the innovative integration of nutritional and inflammatory markers within a machine learning framework, offering a novel approach to survival estimation in advanced gastric cancer. Beyond immediate clinical relevance, the model provides a foundation for future investigations into biomarker-driven risk stratification and personalised interventions in oncology nutrition and supportive care. These findings may stimulate further validation efforts and inspire the development of advanced predictive tools across related patient populations. In clinical settings, the model could be embedded into routine nutritional risk assessment protocols, helping clinicians to identify patients at increased risk and optimise timing and intensity of supportive care interventions. This may be particularly relevant in multidisciplinary teams managing advanced cancer and home parenteral nutrition pathways.

## 5. Conclusions

The application of the RSF algorithm enabled the development of an accurate prognostic model integrating CONUT and LMR indicators for survival assessment in patients with advanced gastric cancer. The analysis confirmed the prognostic value of both markers, with CONUT demonstrating notably stronger predictive power.

The model may serve as a useful tool for identifying patients at increased risk of poor outcomes due to malnutrition and systemic inflammation, supporting nutritional risk stratification and informing therapeutic strategies in interdisciplinary care. In particular, the model may assist clinical decision-making regarding qualification for home parenteral nutrition and adjustment of treatment intensity, especially in patients with elevated risk of malnutrition and heightened inflammatory response.

The analysis revealed a hierarchical structure in the impact of biomarkers on survival: nutritional status played a dominant role, while immuno-inflammatory factors exerted a modulatory effect, particularly in patients with more severe malnutrition. The model proved especially accurate in predicting survival over the first 12–24 months, which represents a clinically meaningful timeframe in the management of patients with advanced cancer.

Beyond its immediate clinical applicability, the proposed approach constitutes a promising foundation for future research on risk stratification and individualised nutritional interventions in oncology. The use of objective and reproducible indicators reduces the risk of bias associated with retrospective designs and supports the development of prognostic tools that can be validated and implemented across diverse clinical settings.

## Figures and Tables

**Table 1:** Baseline distribution of nutritional and inflammatory markers in patients with stage IV gastric cancer receiving home parenteral nutrition.

Baseline Characteristics of Stage IV Gastric Cancer Patients	*n* = 410
Body mass index (kg/m^2^)	
BMI < 18.5	152 (37)
18.5 ≤ BMI < 25	211(51)
BMI ≥ 25	47 (11)
Systemic immune–inflammation index (cut-off = 758.5)	
SII < 758.5	177 (43)
SII ≥ 758.5	233 (57)
Lymphocyte-to-monocyte ratio (cut-off = 2.078)	
LMR < 2.078	198 (48)
LMR ≥ 2.078	212 (52)
Total lymphocyte count	
TLC < 800	77 (19)
800 ≤ TLC < 1200	80 (20)
1200 ≤ TLC < 1500	65 (16)
TLC ≥ 1500	188 (46)
Neutrophil-to-lymphocyte ratio (cut-off = 3.319)	
NLR < 3.319	259 (63)
NLR ≥ 3.319	151 (37)
Naples Prognostic Score	
Score 0: low risk	56 (14)
Score 1–2: intermediate risk	134 (33)
Score 3–4: high risk	220 (54)
Controlling Nutritional Status Score	
Score 0–1: well-nourished	112 (27)
Score 2–4: mildly malnourished	167 (41)
Score 5–8: moderately malnourished	108 (26)
Score 9–12: severely malnourished	23 (6)
Prognostic nutritional index (cut-off = 34.013)	
PNI < 34.013	252 (61)
PNI ≥ 34.013	158 (39)

Data are presented as number and percentage (*n*, %). BMI, body mass index; PNI, prognostic nutritional index; LMR, lymphocyte-to-monocyte ratio; NLR, neutrophil-to-lymphocyte ratio; SII, systemic immune–inflammation index; TLC, total lymphocyte count. Cut-off values used for dichotomisation are shown in parentheses.

**Table 2:** Presents the AUC values, optimal cut-off values, and corresponding sensitivities and specificities for the four analysed biomarkers.

Variables	AUC	Cut-Off Value	Sensitivity	Specificity
Prognostic Nutritional Index	0.744	34.013	0.818	0.585
Lymphocyte-to-Monocyte Ratio	0.678	2.078	0.680	0.643
Neutrophil-to-Lymphocyte Ratio	0.678	3.319	0.517	0.780
Systemic Immune–Inflammation Index	0.653	758.5	0.561	0.693

AUC, area under the receiver operating characteristic curve. Cut-off values were determined using Youden’s index. Sensitivity and specificity refer to discrimination of survival status at the defined threshold.

**Table 3:** Univariate and multivariate Cox regression for overall survival in stage IV gastric cancer with home parenteral nutrition.

Variables	Univariate Analysis			Multivariate Analysis		
		HR (95% CI)			HR (95% CI)	
	HR	Lower–Upper	*p*-Value	HR	Lower–Upper	*p*-Value
Age (years)	1.002	0.993–1.011	0.640			
Gender (Female vs. Male)	1.184	0.890–1.574	0.102			
BMI (kg/m^2^)	0.943	0.844–1.054	0.304			
Systemic Immune–Inflammation Index	0.663	0.554–0.792	<0.001	1.076	0.809–1.431	0.613
Lymphocyte-to-Monocyte Ratio	0.487	0.428–0.555	<0.001	0.632	0.514–0.777	<0.001
Total Lymphocyte Count	0.739	0.650–0.839	<0.001	0.982	0.871–1.109	0.778
Neutrophil-to-Lymphocyte Ratio	1.807	1.538–2.124	<0.001	1.352	0.998–1.831	0.051
Naples Prognostic Score	1.558	1.346–1.802	<0.001	1.059	0.889–1.260	0.519
Controlling Nutritional Status Score	2.110	1.838–2.424	<0.001	1.656	1.306–2.101	<0.001
Prognostic Nutritional Index	0.403	0.338–0.480	<0.001	0.743	0.569–0.970	0.029

HR, hazard ratio; CI, confidence interval; BMI, body mass index. Variables with *p* < 0.001 were considered statistically significant.

**Table 4:** Correlation matrix and Variance Inflation Factor for prognostic biomarkers.

Variables	SII	LMR	TLC	NPS	CONUT	PNI	NLR	Variance Inflation Factor
SII	1.000	0.202	0.372	−0.204	−0.352	0.210	−0.702	1.992
LMR	0.202	1.000	0.297	−0.273	−0.398	0.298	−0.254	1.247
TLC	0.372	0.297	1.000	−0.177	−0.592	0.243	−0.459	1.938
NPS	−0.204	−0.273	−0.177	1.000	0.559	−0.455	0.240	1.582
CONUT	−0.352	−0.398	−0.592	0.559	1.000	−0.693	0.429	3.828
PNI	0.210	0.298	0.243	−0.455	−0.693	1.000	−0.247	2.127
NLR	−0.702	−0.254	−0.459	0.240	0.429	−0.247	1.000	2.226

SII, systemic immune–inflammation index; LMR, lymphocyte-to-monocyte ratio; TLC, total lymphocyte count; NPS, Naples Prognostic Score; CONUT, Controlling Nutritional Status; PNI, prognostic nutritional index; NLR, neutrophil-to-lymphocyte ratio. Variance Inflation Factor values below 5 indicate absence of multicollinearity.

**Table 5:** Estimated survival probabilities by CONUT and LMR categories.

CONUT Category	LMR Category	6 m	12 m	18 m	24 m	30 m	36 m	42 m
1	1	100.0%	82.8%	68.9%	53.3%	38.1%	26.8%	22.6%
1	0	100.0%	75.9%	52.8%	41.1%	28.9%	23.7%	17.5%
2	1	95.4%	70.0%	43.4%	31.1%	22.2%	13.3%	8.8%
2	0	88.5%	53.3%	32.6%	23.5%	16.0%	10.2%	5.8%
3	1	61.4%	31.4%	26.7%	22.5%	13.4%	8.7%	4.4%
3	0	33.4%	12.7%	3.0%	1.5%	1.5%	1.5%	1.5%
4	1	60.5%	29.7%	4.7%	3.9%	2.4%	1.5%	0.7%
4	0	13.6%	0.2%	0.0%	0.0%	0.0%	0.0%	0.0%

Survival probabilities at 6 to 42 months estimated using the Random Survival Forest model across combined categories of Controlling Nutritional Status (CONUT) score and lymphocyte-to-monocyte ratio (LMR). CONUT, Controlling Nutritional Status; LMR, lymphocyte-to-monocyte ratio; m, month.

## Data Availability

The data presented in this study are available on request from the corresponding author for any academic use upon citation of this article. The data are not publicly available due to privacy and permission constraints that restrict their use to the publication of this article only.

## Abbreviations

The following abbreviations are used in this manuscript:

AUC	Area Under the Receiver Operating Characteristic Curve
BMI	Body Mass Index;
CONUT	Controlling Nutritional Status
C-index	Harrell’s Concordance Index
HR	Hazard Ratio
HPN	Home Parenteral Nutrition
LMR	Lymphocyte-to-Monocyte Ratio
NLR	Neutrophil-to-Lymphocyte Ratio
NPS	Naples Prognostic Score
PNI	Prognostic Nutritional Index
ROC	Receiver Operating Characteristic Curve
RSF	Random Survival Forest
SII	Systemic Immune–Inflammation Index
TLC	Total Lymphocyte Count
VIF	Variance Inflation Factor
